# tPA‐GFP is a reliable probe for detecting compound exocytosis in human pancreatic β‐cells

**DOI:** 10.1096/fba.2024-00131

**Published:** 2024-12-27

**Authors:** Aishwarya A. Makam, Shruti Sharma, Prajwal Nagle, Nandhini M. Sundaram, Vidya Mangala Prasad, Nikhil R. Gandasi

**Affiliations:** ^1^ Department of Developmental Biology and Genetics (DBG) Indian Institute of Science (IISc) Bengaluru Karnataka India; ^2^ Molecular Biophysics Unit Indian Institute of Science Bengaluru Karnataka India; ^3^ Center for Infectious Disease Research Indian Institute of Science Bengaluru Karnataka India; ^4^ Department of Medical Cell Biology Uppsala University Uppsala Sweden

**Keywords:** exocytosis, insulin granules, large dense core vesicles, total internal reflection fluorescence microscopy

## Abstract

Pancreatic β‐cells secrete insulin stored in large dense core vesicles (LDCV) by fusion of vesicle and plasma membrane during a process called insulin exocytosis. Insulin secretion is biphasic with a fast first phase and a sustained second phase. Previous studies have pointed out that exocytosis of insulin can occur via (1) single LDCVs fusing with the plasma membrane to release their content or (2) multiple vesicles are involved during a process called compound exocytosis. Compound exocytosis represents a specialized form of secretion in which vesicles undergo homotypic fusion either before (multi‐vesicular exocytosis) or continuous fusion in a sequential manner from (sequential exocytosis) within the same site at the plasma membrane. We see that the number of multi‐vesicles is few and not localized in the vicinity of the plasma membrane. Studying the kinetics of this process and correlating it with biphasic insulin secretion is not possible since there are no specific probes to detect them. It is challenging to identify compound exocytosis with probes that exist for simple exocytosis. To advance our understanding, we need a fluorescent probe that could detect secretory vesicles undergoing compound exocytosis and allow us to distinguish it from other modes of exocytosis. Here, we used two cargo proteins (NPY and tPA) labeled with different fluorescent proteins (mCherry GFP and eGFP) and employed total internal reflection fluorescence microscopy (TIRF‐M) to capture distinct single‐granule and multi‐granular fusion events. We identified tPA‐GFP as a better probe for studying compound exocytosis, as it can detect both simple and sequential exocytosis reliably. Using these probes, we have studied the kinetics of compound exocytosis in human β‐cells. These observations, with additional experiments, may open a whole new field to study the impact of compound exocytosis on biphasic secretion of insulin. Identifying targets to increase the compound exocytosis process can help potentiate insulin secretion in diabetics.

## Graphical abstract

Tissue plasminogen activator tagged to GFP (tPA‐GFP) specifically enables the labelling of insulin granules, a type of large dense‐core vesicle of the pancreatic β cells. When compared to other markers widely used to understand the behavior of insulin granules, tPA enabled the detection of the most number of simple (blue arrow) and compound exocytotic (gray arrows) events usingtotal internal fluorescence reflection microscopy (TIRF microscopy). This makes it a valuable tool in the understanding of the depths of compound exocytosis, including its mechanics and the machinery involved.

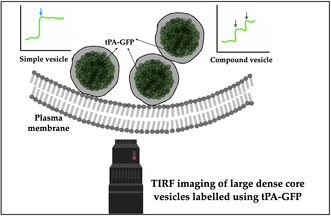

## 1 INTRODUCTION

Exocytosis is a process that aids in cell‐to‐cell communication and occurs in all secretory cells including the cells of the nervous and the endocrine systems.^1^ Exocytosis involves the release of vital chemical messenger molecules like hormones, neurotransmitters and peptides, stored and packed in compact structures called as vesicles.^2^ During exocytosis, secretory vesicles (SVs) loaded with cargo undergo fusion with the plasma membrane (PM) to release their contents into the extracellular environment.^3^ Cells employ various forms of exocytosis to regulate the rate and amount of vesicular content release. It can progress by various mechanisms, which include simple exocytosis, kiss‐and‐run exocytosis, cavicapture, piecemeal degranulation and compound exocytosis.^4^

Compound exocytosis, is characterized by the fusion of SVs with each other and the plasma membrane. It is of two types: sequential exocytosis and multi‐vesicular exocytosis. Sequential exocytosis involves the selective fusion of vesicles with other vesicles that have previously fused with the plasma membrane. On the other hand, multi‐vesicular exocytosis involves the fusion of several vesicles before their fusion with the plasma membrane.^5^ Pancreatic acinar cells and mast cells are the major cell types that have been reported to show greater percentages of compound exocytosis. However, compound exocytosis in pancreatic beta cells accounts for only 2% of total insulin secretory granule (ISG) exocytosis.^6^ They have gained recent importance and are known to account for around 20% of total ISG exocytosis when stimulated by Glucagon‐like peptide 1 and by overexpressing Munc18b.^7^, ^8^ How this pertains to the energy expenditure of the pancreatic β‐cells during biphasic secretion, needs to be explored. A major drawback in this area has been the quest to find a technique suitable for β‐cells that simultaneously allows for real‐time monitoring of these complex events.

Electron microscopy, capacitance measurements and fluorescence microscopy have been the major and feasible ways of detecting exocytosis. In this study, we have employed fluorescence microscopy and assessed the obtained results using electron microscopy, for detecting compound exocytosis. Fluorescently tagged neuropeptide Y (NPY) and tissue plasminogen activator (tPA) are cargoes that can enter the large dense core vesicles (LDCVs), and have thus been used as probes to detect exocytosis.^9^, ^10^ Here, we have surveyed three probes: NPY‐eGFP, NPY‐mCherry and tPA‐GFP,^11^ (that are generally used for detection of simple exocytosis), for the detection of compound exocytosis. Compound exocytosis is more challenging to monitor than simple exocytosis as it involves multiple vesicles releasing their contents at once, making it difficult to differentiate between them kinetically. To track this dynamic event, constant monitoring is needed and this was accomplished by total internal reflection fluorescence‐based live cell imaging. By employing these probes, we have been able to detect compound exocytosis using tPA‐GFP.

We further characterize the distribution of compound exocytosis in β‐cells by exploiting the tPA‐GFP probe to kinetically characterize compound exocytosis.

## 2 MATERIALS AND METHODS

### 2.1 Cell culture

INS1 832/13 cells were cultured in RPMI 1640 (Invitrogen) supplemented with 10% fetal bovine serum (FBS), streptomycin (100 U/mL), penicillin (100 U/mL), sodium pyruvate (1 mM), and 2‐mercaptoethanol (50 μM).

Transient transfections were performed on 22 mm poly‐L‐lysine‐coated coverslips in 100 μL OptiMEM (Invitrogen) using 0.5 μL Lipofectamine® 2000 (Invitrogen), 0.2–0.6 μg plasmid DNA. The reaction was terminated after 3–5 h, and imaging was performed 24–48 h after transfection.

### 2.2 Constructs

The constructs used in this study were Neuropeptide Y (NPY)‐mCherry, (kindly provided by Sebastian Barg), NPY‐eGFP and tPA‐GFP^11^ (kind gift from Sebastian Barg).

### 2.3 Solutions

Cells were imaged in a solution containing 138 mM sodium chloride (NaCl), 5.6 mM potassium chloride (KCl), 1.2 mM magnesium chloride (MgCl_2_), 2.6 mM calcium chloride (CaCl_2_), 3 mM D‐glucose, 5 mM HEPES pH 7.4 with 1 M sodium hydroxide (NaOH).

For exocytosis of ISGs, the buffer instead contained 10 mM glucose and was supplemented with 2 mM forskolin and 200 μM diazoxide, a K^+^ ATP channel opener that prevents glucose dependent depolarization. Exocytosis was then evoked by computer‐timed local application of high K^+^ (75 mM KCl equimolarly replacing NaCl) through a pressurized glass electrode similar to those used for patch clamp experiments.

### 2.4 Microscopy

Cells were imaged using a total internal reflection (TIRF) microscope based on an AxioObserver Z1 with a 100×/1.45 objective (Carl Zeiss, Jena, Germany). Excitation was from two DPSS lasers at 491 and 561 nm or individual lasers as described in individual experiments. The emission light was chromatically separated into separate areas of an EMCCD camera (Photometrics Evolve) using an image splitter (Photometrics DV2, Photometrics, Tucson, AZ, USA). Alignment of the two‐color channels was corrected.

### 2.5 Image analysis

Image analysis was done using Metamorph software. The images were scanned manually for individual simple and compound exocytosis events. All vesicles undergoing exocytosis were identified by drawing a 7 × 7 pixel circle (region of interest, ROI) centered on the brightest pixel of the first frame indicating exocytosis, and their corresponding location and timestamps were also recorded. Exocytotic events were distinguished from the undocking as sudden disappearance/appearance of the vesicle's fluorescence, rather than the slow disappearance observed in the fluorescence of an undocking vesicle^12^. The first frame exhibiting a discernible rise or fall in the granule's fluorescence was defined as the onset of exocytosis. The average pixel fluorescence in (i) a central circle (c) with a diameter of 3 pixel (0.5 m), (ii) an annulus (a) with an outer diameter of 5 pixel (0.8 m) and (iii) a background (bg) area excluding any cell was then read using an algorithm written on Metamorph journal. For each frame, the average backdrop outside the cell area (bg) was first subtracted. The value of the annulus (a) was consequently subtracted from the value of the circle (c) to get the specific granule's fluorescence Δ*F* (Δ*F* = *c* − *a*). Simple and compound events' Δ*F* versus time data was recorded and plotted in an Excel sheet enabling the plotting of graphs.

Vesicles undergoing exocytosis were classified and quantified based on their localization in the cell. The edge of the cell was marked manually. Then, another boundary approx. eight to ten pixels from the border was marked inside of it. The vesicles between the border and the inner boundary were classified as peripheral. The vesicles within the inner border were classified as central vesicles.

### 2.6 Sample preparation for ultramicrotomy

INS‐1 832/13 cells were grown in 6‐well plates in RPMI 1640 medium supplemented with 11.1 mM glucose, 10% fetal bovine serum (FBS), 100 U/mL penicillin–streptomycin, 1 mM sodium pyruvate, 2 mM glutamine and 50 μM 2‐mercaptoethanol, at 37°C and 5% CO_2_. When confluent, the cells were trypsinized and harvested followed by centrifugation at 3000 rpm for 5 min at room temperature to obtain 0.5–1 mm^3^ (~10^6^ cells or more) pellets. The pellets were subjected to double fixation with fresh 2.5% glutaraldehyde‐2% paraformaldehyde (prepared in 0.1 M sodium cacodylate buffer) for 2 h and 1% osmium tetroxide (OsO_4_) for 1 h at room temperature. Pellets were washed three times between the fixatives with cacodylate buffer. For enhanced contrast, the pellets were immersed in 2% uranyl acetate (prepared in 50% ethanol) for 1 h. They were then subjected to dehydration through a graded series of ethanol concentrations (75%, 95% and 100%). LR white resin (TedPella Inc.) diluted (1:1) with 100% ethanol was allowed to infiltrate the pellets for 1 h at room temperature. The resin embedding was done in plastic BEEM capsules (TedPella Inc.) for 48 h at 65°C.

### 2.7 Ultramicrotomy and transmission electron microscopy

Ultrathin sections of 50–100 nm thickness were obtained using glass knives on Leica EM UC7 Ultramicrotome and collected on 300‐mesh copper grids. The sections were stained with 2% uranyl acetate for 30 min at room temperature. The images were then captured in Talos L120C transmission electron microscope at 22000X (6.40 Å/pixel).

### 2.8 Statistics

Data is presented as mean ± SEM unless otherwise stated. All the data was tested for statistical significance using Students *t*‐test for one‐tailed, unpaired samples, as appropriate.

## 3 RESULTS

### 3.1 Visualization of LDCVs using electron microscopy images

**FIGURE 1:**
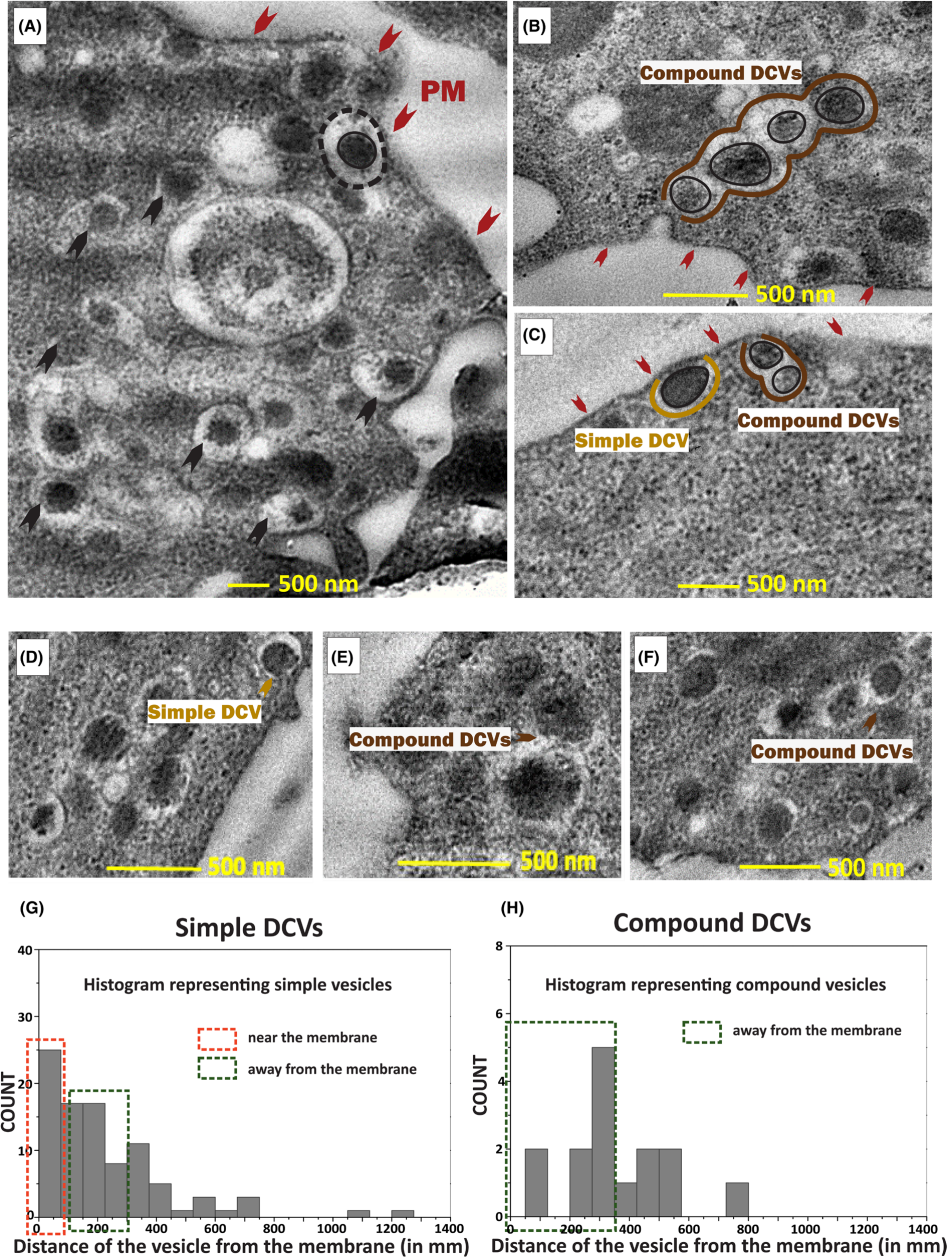
TEM images of sections of INS −1 cells showing vesicles undergoing both simple and compound exocytosis: (A) A section of INS −1 cell showing vesicles undergoing simple and compound exocytosis. The plasma membrane is indicated using red arrows. A representative LDCV is annotated with the vesicle membrane shown as dotted marking and the dense core of the vesicle has been encircled in a solid line (B, E, F) Vesicle undergoing compound exocytosis/Compound dense core vesicles (Compound DCVs). Red arrows indicate the plasma membrane, the brown markings and arrows indicate the vesicle membrane (DCV‐ dense core vesicle). Black solid markings in (B) indicate the dense core. (C, D) Vesicle undergoing simple exocytosis/Simple dense core vesicles (Simple DCVs). A representative simple DCV membrane has been outlined in yellow and the dense core in black in (C, G, H) Histogram of the number of vesicles undergoing simple and compound exocytosis (simple DCVs and compound DCVs) and their distance from the plasma membrane.

INS‐1 cells, a widely used beta cell line, were resin‐embedded and sectioned at room temperature using an ultramicrotome. These cell sections were then imaged using transmission electron microscopy (TEM). Figure 1A indicates a representative TEM image capturing the presence of simple and compound fusion events of LDCVs near the plasma membrane. The LDCVs undergoing simple exocytosis are visible as single and circular among other vesicles and are labeled as simple dense core vesicles (Simple DCV) (Figure 1C,D). The ones undergoing compound exocytosis have two or more vesicles sticking together as shown in Figure 1B,E,F and are marked as compound dense core vesicles (Compound DCVs). Figure 1G,H represent histograms indicating that LDCVs undergoing simple exocytosis mostly occur close to the membrane (bars inside the dotted red box) and the ones undergoing compound exocytosis are farther away from the membrane (bars inside the dotted green box). Under basal conditions, these cells show a large number of LDCVs that correspond to vesicles undergoing both simple and compound exocytosis, making this cell‐line a good model to study compound exocytosis. A more dynamic technique like fluorescence‐based live cell imaging has been used to understand the intricate details of these two visibly different types of LDCV fusion and release.

### 3.2 Detection of simple exocytosis in Ins‐1 cells

**FIGURE 2:**
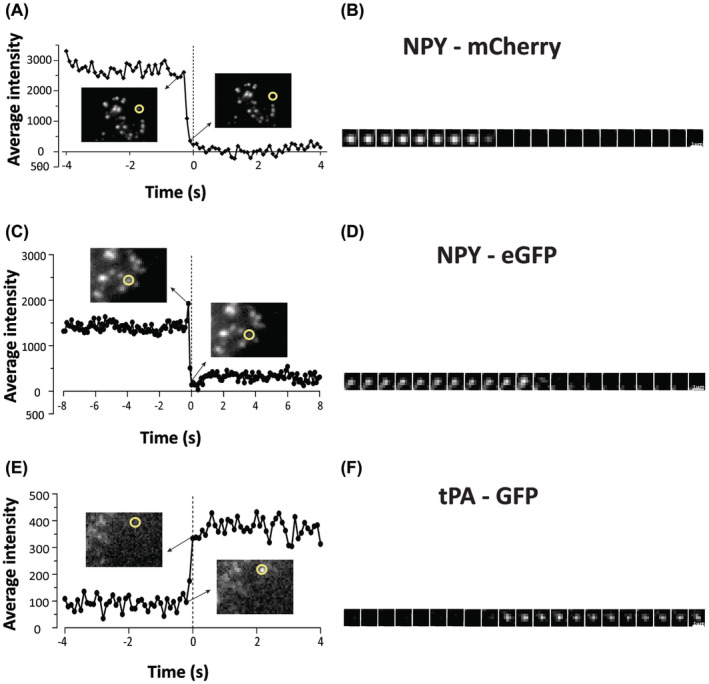
Simple exocytosis events detected using three probes: NPY‐mCherry, NPY‐eGFP and tPA‐GFP. (A, C, E) Intensity vs. time plots—Flourescence intensity of a NPY‐mCherry, NPY‐eGFP, and tPA‐GFP labeled granules undergoing simple exocytosis plotted against time respectively (Detection of intensity has been explained in the methods section). (B, D, F) Montages representing a single NPY‐mCherry, NPY‐eGFP, and tPA‐GFP labeled granule undergoing simple exocytosis respectively.

Here we exploit, labelling LDCVs by introducing fluorescent labels in the cargo to visualize exocytosis. Fluorescently tagged NPY and tPA used here are the appropriate cargo molecules that are part of LDCVs of INS‐1 cells. Exocytosis was stimulated by exposing the cells to elevated K^+^ by KCl stimulation, as described in the methodology. Figure 2A,C represents the intensity time plot of the secretory vesicles labeled using NPY‐mCherry and NPY‐eGFP. After the secretory vesicle fuses with the plasma membrane, its fluorescence suddenly falls in a few microseconds before it completely disappears (marked at zero seconds), signifying that the fluorophore attached NPY is released from the cells. Figure 2B,D represent this process with the help of a montage showing a single secretory vesicle undergoing simple exocytosis.

Although NPY is well suited to report exocytosis, it leaves the granule quickly. tPA, a 70 kDa serine protease, has been reported to leave the granules much more slowly, and hence it could prove to be a better probe for studying exocytosis. A fluorescence‐time plot and a montage of a single tPA‐GFP tagged SV undergoing exocytosis are shown in Figure 2E,F. As compared to NPY‐eGFP/mCherry‐tagged SVs, the tPA‐GFP‐tagged SVs were very faintly visible until they fused with the PM. After the fusion, instead of suddenly disappearing, the fluorescence decreased gradually over time (not shown in the plot) because tPA is retained much longer than NPY and was slowly released outside.

### 3.3 Characterization of simple and compound exocytosis

**FIGURE 3:**
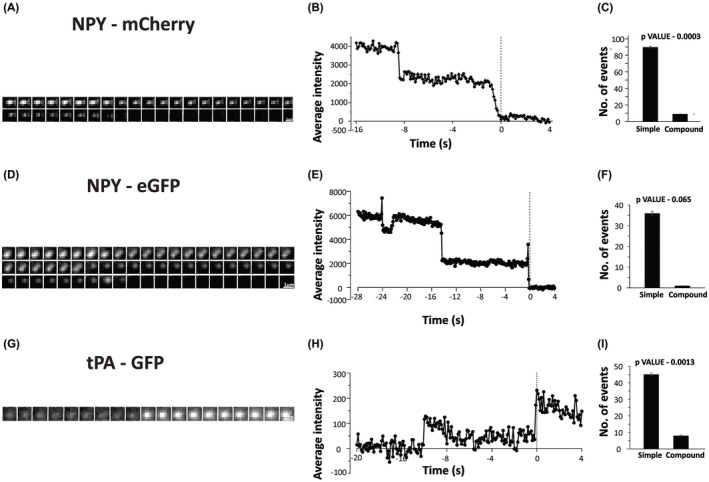
Detection of compound exocytosis using NPY‐mCherry, NPY‐eGFP, and tPA‐GFP. (A, D, G) Montages representing a single NPY‐mCherry, NPY‐eGFP, and tPA‐GFP labeled granules undergoing compound exocytosis respectively. (B, E, H) Intensity versus time plots representing the compound exocytosis using the three probes respectively (Detection of intensity has been explained in the methods section). (C, F, I) Bar graphs representing the number of simple and compound exocytosis events that were observed using each of the probe mentioned above.

Further, we employed TIRF microscopy to visualize and distinguish simple exocytosis and compound exocytosis using the fluorescent probes described earlier. Simple exocytosis was characterized by typical single flash events indicating the fusion of single SVs with the PM (Figure 2). In addition to this, we observed multiple flashes combined with flashes of longer duration and categorized them as compound exocytosis events (Figure 3A,D,G). The corresponding intensity versus time plots are shown in Figure 3B,E,H, respectively. The number of these events were quantified by calculating the total number of fusion events for each of the probes and the percentage of simple and compound events. The number of simple and compound events were counted using all the 3 probes and bar graphs were plotted (Figure 3C,F,I) to understand the frequency of their occurrence. Compound exocytosis events were detected in cells transfected with all the three probes although the frequency of detection was higher with tPA‐GFP.

### 3.4 Localization of SVs undergoing simple and compound exocytosis

**FIGURE 4:**
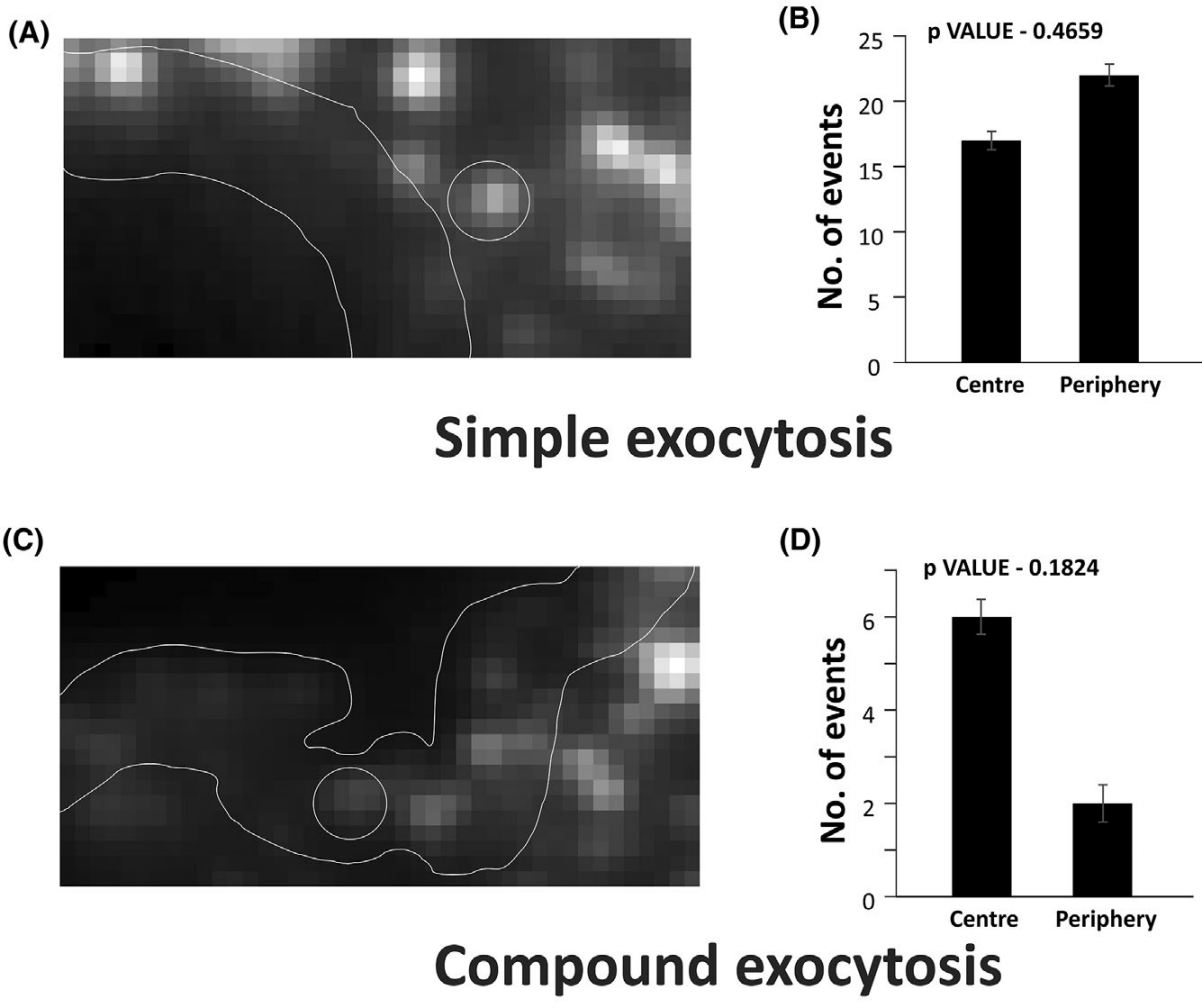
Spatial localisation of tPA‐GFP labeled vesicles undergoing simple and compound exocytosis. (A, C) Image of the cell representing the marked cell boundary and the inner boundary. This was defined for both simple and compound exocytosis events respectively. (B, D) A bar graph representing the number of simple and compound exocytosis events occurring at the centre and the periphery of the cell.

Further, the localization of the release events was studied. Although TIRF microscopy marks only a footprint of the cell, polarity‐based observations can be made. To do this we first, divided the TIRF image of the cell into central and peripheral (8–10 pixels from the cell boundary) regions, as shown in Figure 4A,C.

Fusion events using tPA‐GFP was identified as simple and compound exocytosis respectively. They were further classified into centre and periphery, based on their localization in the cell. Of the total number of simple events, the events happening near the centre and periphery were estimated. (Figure 4B,D). These results show a pattern wherein simple events appear to be more localized in the periphery of the cell, representing the plasma membrane and approximately 70–80 nm below the plasma membrane. In contrast, compound events are found to be occurring in the centre of the cell, comprising mainly the cytoplasmic region.

## 4 DISCUSSION

Exocytosis is defined as the fusion of membrane‐enclosed vesicles with the plasma membrane leading to the subsequent release of the cargo into the extracellular milieu. Two types of exocytosis have been shown to occur in secretory cells viz. (a) simple exocytosis where a single vesicle fuses with the membrane, and (b) compound exocytosis—where more than one vesicle is involved in the fusion process. The additional step with compound exocytosis is vesicle‐to‐vesicle fusion which enhances secretory output.^13^ Compound exocytosis is an energy‐efficient way for a cell to release large amounts of content with limited expenditure. During simple exocytosis, repeated rounds of granule fusion followed by endocytosis of the fused granules would become necessary.^14^ This load on the cell is considerably reduced by employing compound exocytosis. What remains unexplored is the situations in which the cell would employ compound exocytosis.

Secretory granule exocytosis primarily in neuroendocrine cells like β‐cells, suggests the presence of 2 pools of granules in cells viz. (i) the readily releasable pool, and (ii) the reserve pool.^15^, ^16^, ^17^, ^18^, ^19^ The readily releasable pool is the one that consists of vesicles docked at the active zone and primed for release whereas the reserve pool consists of vesicles that are further away from the membrane and replenish the readily releasable pool upon stimulation.^16^, ^20^ In β‐cells, the secretion of insulin present in these granules is controlled by glucose in a biphasic model. The first phase is the rapid phase lasting for 5–10 min, followed by a second phase of slow and pulsatile release.^21^, ^22^, ^23^ These two pools of insulin granules differ enormously in their release probabilities which depends on the molecular machinery associated with them and their distance from the plasma membrane. At a given time, a β‐cell has around 6000–10,000 insulin granules, out of which only 600–1000 of them are release‐ready.^24^, ^25^ To be release‐ready, they get transported to the plasma membrane and, dock to the membrane, which is followed by the assembly of the molecular machinery during a process called priming, leading to exocytosis. Once the cell is stimulated, the replenishment of the docked granules is thought to sequentially restore the lost pool of primed granules.^24^, ^26^, ^27^ It has been previously shown that docking of new granules to the plasma membrane is the rate‐limiting step. The sequential maturation model requires repeated docking and further priming for a sustained second phase of release. This is because the first phase of release depletes the docked granules. Compound exocytosis, thus, might be an energy‐efficient process so that repeated docking can be avoided. On the contrary, we observe that the vesicles showing compound fusion behavior, are present further away from the membrane (>200 nm from the membrane). This is probably the reason why the cell adopts sequential recruitment to facilitate simple exocytosis for sustained release during the second phase. Compound exocytosis of granules likely enables kinetically compensating for the time required for granules to dock and prime for the second phase of release. This would be very beneficial concerning the amount of insulin that is being transported to the membrane in the form of compound vesicles when compared to simple ones. This reduces the time required to meet the demands of further release at the membrane.

To understand these concepts in greater depth, the kinetics or dynamics of compound exocytosis have to be extensively explored. To understand the kinetics of compound exocytosis, it is important to evaluate compound exocytosis in live human β‐cells for extended periods. Fluorescent‐based live cell microscopy makes this possible but existing probes do not complement this. We tested various markers that can label compound exocytosis events in live human β‐cells. We came across tPA‐GFP as an able candidate. tPA‐GFP has the ability to detect simple and compound events differentially. NPY (Neuropeptide Y), a cargo of large dense‐core vesicles, is one of the widely used markers for large dense‐core vesicle exocytosis. In comparison to these established markers, tPA‐GFP was able to efficiently detect simple as well as compound exocytotic events. In the case of, NPY‐mCherry and NPY‐eGFP, the sudden loss of fluorescence was an indication of an exocytotic event, whereas with tPA‐GFP, when exocytosis occurs, tPA remains stuck to the membrane enabling the observation of post‐exocytotic events. Here, we have taken advantage of this nature of tPA, to differentiate between simple and compound exocytotic events. Overall, this probe provides an opportunity to understand the depths of compound exocytosis and its kinetics in detail. Additional experiments using the probe, will allow exploration of how human β‐cells can employ compound exocytosis during the first and second phases of insulin secretion to mitigate high energy requirements. This might have implications in ER‐stress mediated changes during type‐1 diabetes^28^ and type‐2 diabetes where different therapeutic strategies including GPCR,^29^ GLUT mediated pathways are being explored.^30^, ^31^

## AUTHOR CONTRIBUTIONS

Conceptualization—N.R.G; Methodology—V.M.P. and N.R.G.; Validation—A.A.M., S.S., P.N., N.M.S., V.M.P. and N.R.G.; Formal analysis—A.A.M., P.N., V.M.P. and N.R.G.; Investigation—A.A.M., V.M.P. and N.R.G.; Resources—V.M.P. and N.R.G.; Data curation—A.A.M., S.S., P.N., N.M.S., V.M.P. and N.R.G.; Writing—A.A.M., V.M.P. and N.R.G.; Review & editing—A.A.M., S.S., P.N., N.M.S., V.M.P. and N.R.G.; Supervision—A.A.M., V.M.P. and N.R.G.; Project administration—V.M.P. and N.R.G.; Funding acquisition—N.R.G. All authors have read and agreed to the published version of the manuscript.

## FUNDING INFORMATION

This research was funded by the Indian Institute of Science—seed grants, Department of Biotechnology (DBT)‐Ramalingaswami fellowship, Indian Council of Medical Research (ICMR)—Grants in Aid Scheme, Department of Science and Technology (DST)—Science and Engineering Research Board (SERB)—Starting grants, Longevity India Initiative and Novo Nordisk Foundation grant awarded to NRG lab. This work was also supported by the Infosys Young Investigator fellowship to VMP and NRG. AAM was supported by a fellowship from DBT and the Prime Ministers Research Fellowship (PMRF) given for pursuing her PhD. SS was supported with a fellowship from the Indian Institute of Science for pursuing her PhD. PN was supported with an Innovation in Science Pursuit for Inspired Research (INSPIRE) fellowship by DST. NM was supported by a fellowship from DBT for pursuing her PhD. DST‐FIST supported facilities in the department contributed to this work.

## CONFLICT OF INTEREST STATEMENT

The authors declare that the research was conducted in the absence of any commercial or financial relationships that could be construed as a potential conflict of interest.

## Data Availability

Included in article. The data that support the findings of this study are available in the Materials and Methods, Results, and/or Supplemental Material of this article. If there are any further requests regarding datasets, it would be made available upon reasonable request.
